# Prevalence of Prediabetes and Undiagnosed Diabetes Among Kuwaiti Adults: A Cross-Sectional Study

**DOI:** 10.2147/DMSO.S296848

**Published:** 2021-05-17

**Authors:** Anwar Mohammad, Ali H Ziyab, Talal Mohammad

**Affiliations:** 1 Department of Biochemistry and Molecular Biology, Dasman Diabetes Institute, Kuwait City, Kuwait; 2 Department of Community Medicine and Behavioral Sciences, Faculty of Medicine, Kuwait University, Safat, Kuwait; 3 St. Antony’s College, University of Oxford, Oxford, UK; 4 Department of Biological Anthropology, University of Cambridge, Cambridge, UK

**Keywords:** prevalence, diabetes mellitus, prediabetes, undiagnosed, Kuwait

## Abstract

**Purpose:**

This study aimed to estimate the prevalence of prediabetes and undiagnosed type 2 diabetes mellitus (T2DM) according to fasting plasma glucose (FPG), 2-h plasma glucose (PG) during oral glucose tolerance test (OGTT), and glycated hemoglobin (HbA1c) among a sample of Kuwaiti adults. In addition, associations of prediabetes and undiagnosed T2DM with sex, age, and body mass index (BMI) were assessed.

**Methods:**

A cross-sectional study enrolled 1238 subjects aged 18–65 years who reported no prior history of DM. After overnight fasting, FPG and HbA1c were measured in the total study sample, and 2-h PG during a 75-g OGTT was measured in a subsample of 155 subjects. Prediabetes and undiagnosed T2DM were defined according to the American Diabetes Association criteria. Associations were assessed using Poisson regression with robust variance estimation, and adjusted prevalence ratios (aPRs) and 95% confidence intervals (CIs) were estimated.

**Results:**

We enrolled a total of 618 males and 620 females, with an average age of 43.0 years. The prevalence of prediabetes was estimated to be 47.9% (588/1228) by FPG, 36.8% (57/155) by 2-h PG OGTT, and 31.0% (355/1144) by HbA1c. The prevalence of undiagnosed T2DM was 6.9% (85/1228) by FPG, 11.0% (17/155) by 2-h PG OGTT, and 4.9% (56/1144) by HbA1c. Sex-related differences in the prevalence of prediabetes and undiagnosed T2DM were observed. Prediabetes and undiagnosed T2DM prevalence estimates showed increasing trends as age and BMI increased. For instance, obese subjects compared to under/normal weight subjects had an increased HbA1c-defined prediabetes prevalence (aPR = 1.62, 95% CI: 1.21–2.16).

**Conclusion:**

Prediabetes and undiagnosed T2DM affect a considerable proportion of Kuwaiti adults, and variations across sex, age, and BMI exist. Hence, early identification and management of affected individuals may help reduce the public health burden.

## Introduction

Diabetes mellitus (DM), a significant global public health concern, is a disorder of glucose metabolism that has a substantial impact on the well-being of affected individuals as well as their families and societies. The International Diabetes Federation (IDF) estimated in 2019 that around 463 million people (prevalence: 9.3%) globally are living with DM, with this number expected to rise by 2045 to approximately 700 million people (prevalence: 10.9%).^1^ The economic impact of DM is demonstrated by the high global estimate of direct health expenditure that amounted to 760 billion USD in 2019 and is projected to reach 845 billion USD by 2045.^2^ Prediabetes (intermediate hyperglycemia) and undiagnosed type 2 DM (T2DM) add to the health and economic burden associated with DM. Prediabetes, a transitional, high-risk stage for the development of T2DM, carries microvascular and macrovascular risks to affected individuals.^3–6^ The global prevalence estimate of prediabetes, measured as impaired glucose tolerance (IGT), was reported to be 7.5% in 2019 and is expected to increase to 8.6% in 2045.^1^ Moreover, numerous cases of T2DM are undiagnosed or undetected for several years, during which many complications may develop. Globally, it is estimated that, on average, 50% (range: 24.1% to 75.1%) of people living with diabetes are unaware of their condition.^1^,^7^ As such, individuals with prediabetes and undiagnosed T2DM represent a public health challenge and missed opportunities to prevent complications.

Differences in lifestyle and environmental factors, in addition to genetic susceptibility, account for some of the observed variability in prediabetes and DM prevalence estimates among different populations. For instance, among adults living in the US, the prevalence of DM was estimated to be 14.6% (diagnosed: 10.0%; undiagnosed: 4.6%), and prediabetes affected 37.5% of the study population.^8^ Among Chinese adults, the prevalence of DM was estimated to be 10.9% (diagnosed: 4.0%; undiagnosed: 6.9%), and prediabetes affected 35.7% of the study population.^9^ Out of the seven IDF global regions, the Middle East and North Africa (MENA) region had the highest global age-standardized DM prevalence of 12.2% in 2019.^1^ Kuwait, situated in the MENA region, had an estimated DM prevalence of 22.0% among adults in 2019,^10^ which is well above the global prevalence of 9.3%.^1^ The prevalence of undiagnosed T2DM was estimated to be 4.1% among a sample of working adults in Kuwait.^11^ Moreover, the prevalence of prediabetes was estimated to be 33.3% among adolescents,^12^ 6.3% among young adults,^13^ and 19.4% among adults^14^ in Kuwait. Such elevated prevalence estimates of DM and prediabetes are alarming and place Kuwait among the most highly affected countries worldwide.^15^

In addition to the population characteristics, the diagnostic or screening tests used [fasting plasma glucose (FPG), 2-hour plasma glucose during oral glucose tolerance test (2-h PG OGTT), or glycated hemoglobin (HbA1c)] may contribute to the heterogeneity in prevalence estimates within and between populations. Currently, the burden of prediabetes and undiagnosed T2DM remains scarcely explored in Kuwait, with no prior study reporting prevalence estimates based on FPG, 2-h PG OGTT, and HbA1c. Hence, to better understand the magnitude of prediabetes and undiagnosed T2DM among Kuwaiti adults and to inform healthcare planning and public health preventive strategies, this study sought to estimate the prevalence of prediabetes and undiagnosed T2DM according to FPG, 2-h PG OGTT, and HbA1c criteria among a sample of Kuwaiti adults with no prior history of DM. In addition, associations of prediabetes and undiagnosed T2DM with sex, age, and body mass index (BMI) were assessed.

## Methods

### Study Setting, Design, and Population

Kuwait, a small country with a total area of approximately 18,000 km^2^, is situated on the Arabian Peninsula. Geographically, the country is divided into six governorates. According to the Public Authority for Civil Information, as of June 2017, Kuwait’s estimated population was around 4.4 million, with 1.3 million Kuwaitis (nationals, around 30% of the population), and 3.1 million non-Kuwaitis (about 70% of the population). Of the 1.3 million Kuwaitis, 51% (≈663,000) are female and 49% are male (≈637,000), with 17% of the total population having attained higher education and above. The Kuwait Wellbeing cross-sectional study enrolled 1238 participants aged between 18 and 65 years with no prior history of diabetes from all governorates of Kuwait.^16^ Participation was restricted to individuals of Kuwaiti nationality. The exclusion criteria included pregnancy, known diabetes (prior diagnosis and/or use of diabetes medication), inability to walk unaided, psychosis, or terminal illness. In addition to the general inclusion criteria, participants were invited to undergo the OGTT test if the participant’s measured FPG was <12 mmol/L. Of the total study participants with FPG measurement (n = 1228), three participants had FPG levels ≥12 mmol/L, and hence were not invited to undergo OGTT. The enrollment of subjects started in November 2012 and ceased in October 2017. Given the wide use of mobile phones by the local population, invitations to participate in the study were sent by SMS text messages, using a fixed script describing the study, to random samples of mobile phone subscribers at the three mobile telecommunication providers in Kuwait. SMS text messages were disseminated by a third party that had access to registered mobile phone numbers with the three mobile telecommunication providers in Kuwait. Eligible volunteers attended the Kuwait Wellbeing Unit at the Dasman Diabetes Institute to undergo the study tests and complete the study questionnaires. The protocol of the present study was approved by the Ethical Review Board of the Dasman Diabetes Institute, Safat, Kuwait (RA-01-2010). Written informed consent was obtained from all study participants. The study was conducted in accordance with the principles and guidelines of the Declaration of Helsinki for medical research involving human subjects.

### Biochemical Analysis

Participants were asked to fast overnight for at least 10 h. Upon arrival at the research unit, fasting blood samples were collected, and FPG was tested using a point-of-care HemoCue^®^ Glucose 201 analyzer (HemoCue Inc., Lake Forest, CA). Subsequently, another set of blood samples were collected to measure PG after a 2-h 75-g OGTT. Briefly, volunteers were given 75 g of glucose in a 250-mL solution to drink, and 2-h post consumption, PG was measured using the Siemens Dimension RXL chemistry analyzer (Diamond Diagnostics, Holliston, MA). HbA1c levels were determined using a VariantT M device (BioRad, Hercules, CA).

### Anthropometric Measurements

Height was measured to the nearest 0.1 cm using a stadiometer while weight was measured to the nearest 0.1 kg using a digital scale. Both height and weight were measured without shoes and in light clothing in a standardized manner. Body mass index (BMI) was calculated as weight in kilograms divided by height in meters squared (kg/m^2^). Standard BMI groupings were applied: underweight (BMI < 18.5), normal weight (BMI 18.5–24.9), overweight (BMI 25.0–29.9), and obesity (≥ 30.0).^17^

### Ascertainment of Outcome Variables

Among individuals with no prior history of DM diagnosis and/or no history of using DM pharmacological treatments, prediabetes and undiagnosed T2DM were defined according to the ADA criteria.^18^ The criteria used to define prediabetes according to the different tests are as follows: FPG 5.6–6.9 mmol/L (100–125 mg/dL), 2-h PG 75-g OGTT 7.8–11.0 mmol/L (140–199 mg/dL), and HbA1c 5.7–6.4% (39–47 mmol/mol). Undiagnosed T2DM was defined by the following criteria: FPG ≥ 7.0 mmol/L (126 mg/dL), 2-h PG 75-g OGTT ≥ 11.1 mmol/L (200 mg/dL), and HbA1c ≥ 6.5% (48 mmol/mol).

### Statistical Analysis

Analyses were conducted using SAS 9.4 (SAS Institute, Cary, NC). The statistical significance level was set to α = 0.05 for all association analyses. Descriptive analyses were conducted to calculate the frequencies and proportions of categorical variables and means and standard deviations (SD) of continuous variables. To compare characteristics across the total study sample (n = 1238) and the subsample of individuals who underwent OGTT (n = 155), two-sided one-sample binomial tests were used to compare proportions, and one-sample *t*-tests were used to compare the means of continuous variables. Prevalence estimates of prediabetes and undiagnosed T2DM were estimated and projected to the study population and not the total population of Kuwait. Moreover, chi-squared (*X*^2^) tests were used to assess whether prevalence estimates of prediabetes and undiagnosed T2DM differed across sex, age groups, and BMI categories. When the cell count was less than 5, Fisher’s exact test was used. Adjusted associations were assessed by applying a modified Poisson regression with robust variance estimation using the GENMOD procedure in SAS 9.4 to estimate and infer the prevalence ratios (PRs) and their 95% confidence intervals (CIs).^19^

## Results

A total of 1238 subjects (618 males and 620 females) were enrolled in the current study, and a subsample of participants (n = 155; 89 males and 66 females) agreed to undergo OGTT. The proportion of females in the subsample was lower than the proportion of females in the total study sample (42.6% vs 50.1%, *P* = 0.041; Table 1). The mean age of participants in the total study sample was similar to the mean age of the subjects in the subsample (mean [SD]: 43.0 [11.2] vs 42.1 [9.6] years, *P* = 0.262). In both the total study sample and the subsample, the majority of participants were classified as overweight (43.6% and 42.6%) and obese (37.1% and 39.3%) according to their BMI. On average, there was no difference in BMI between participants in the total study sample and those in the subsample (mean [SD]: 29.2 [6.0] vs 29.3 [5.2] kg/m^2^, *P* = 0.889; Table 1).

**Table 1:** Characteristics of the Total Enrolled Study Sample (n = 1238) and a Subsample with Oral Glucose Tolerance Test (n = 155) Results

Variable	Total Study Sample	Subsample with OGTT Results	*P*-value*
	(n = 1238), % (n)	(n = 155), % (n)	
**Sex**			
Male	49.9 (618)	57.4 (89)	0.062
Female	50.1 (620)	42.6 (66)	0.041
**Age (years)**			
Mean (SD)	43.0 (11.2)	42.1 (9.6)	0.262^†^
≤34	25.1 (311)	25.8 (40)	0.839
35–44	27.3 (338)	33.6 (52)	0.081
45–54	31.0 (384)	30.3 (47)	0.855
≥55	16.6 (205)	10.3 (16)	0.036
**BMI (kg/m^2^)**			
Mean (SD)	29.2 (6.0)	29.3 (5.2)	0.889^†^
Underweight	1.5 (18)	1.3 (2)	0.868
Normal weight	17.8 (221)	16.8 (26)	0.727
Overweight	43.6 (540)	42.6 (66)	0.794
Obese	37.1 (459)	39.3 (61)	0.558

**Notes:** *Two-sided one-sample binomial tests were used to determine if statistical differences were present when comparing proportions of characteristics of the subsample who underwent OGTT with their respective proportions in the total study sample; ^†^calculated using one-sample *t*-tests to compare means of age and body mass index variables in the subsample who underwent oral glucose tolerance test to their respective means in the total study sample.

**Abbreviations:** BMI, Body mass index; SD, standard deviation; OGTT, oral glucose tolerance test.

**Figure 1:**
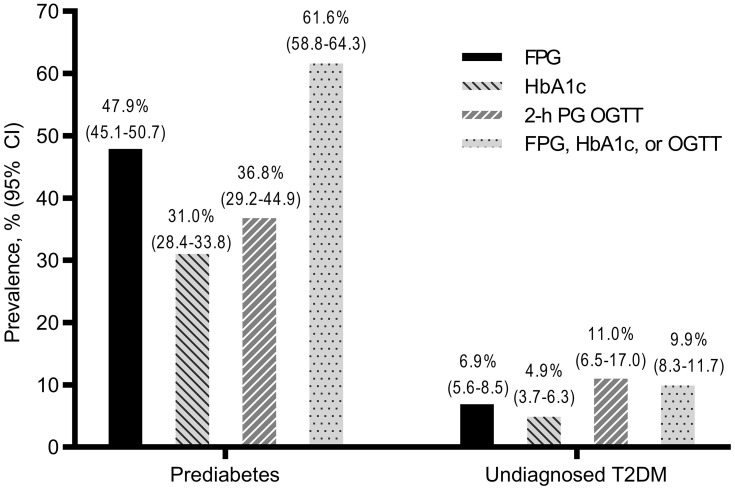
Prevalence estimates of prediabetes and undiagnosed type 2 diabetes mellitus (T2DM) according to fasting plasma glucose (FPG), glycated hemoglobin A1c (HbA1c), 2-h plasma glucose during 75-g oral glucose tolerance test (2-h PG OGTT), and according to FPG, HbA1c, or OGTT. Values plotted above bars represent prevalence % (95% confidence interval [CI]).

Prevalence estimates of prediabetes and undiagnosed T2DM according to FPG, 2-h PG OGTT, HbA1c, and according to any test are shown in Figure 1. The highest prediabetes prevalence was estimated by FPG (47.9%, 588/1228), followed by 2-h PG OGTT (36.8%, 57/155) and HbA1c (31.0%, 355/1144). The highest prevalence estimate of undiagnosed T2DM was reported by 2-h PG OGTT (11.0%, 17/155), followed by FPG (6.9%, 85/1228) and HbA1c (4.9%, 56/1144; Figure 1). Prediabetes prevalence according to any test was estimated to be 61.6% (762/1238), and the prevalence of undiagnosed T2DM according to any test was estimated to be 9.9% (122/1238; Figure 1).

Table 2 shows prevalence estimates of prediabetes identified by different tests according to sex, age groups, and BMI categories. Prediabetes identified by FPG was more common among females than males (51.6% vs 44.1%, *P* = 0.009), whereas prediabetes ascertained by HbA1c was more common among males than females (33.4% vs 28.8%, *P* = 0.042). Similarly, prediabetes defined according to 2-h PG OGTT was more common among males than females (39.3% vs 33.3%, *P* = 0.332), although this difference was not significant. Prediabetes defined according to FPG, HbA1c, and 2-h PG OGTT showed increasing trends as age and BMI increased (Table 2). For instance, the prevalence of FPG-defined prediabetes was estimated to be 59.3% among those aged ≥ 55 years versus 41.2% among those aged ≤ 34 years (aPR = 1.50, 95% CI: 1.26–1.78). Compared to subjects with under/normal weight, participants classified as obese by their BMI had a higher prevalence of FPG-defined prediabetes (aPR = 1.40, 95% CI: 1.17–1.69; Table 2).

**Table 2:** Prevalence Estimates of Prediabetes Measured by Fasting Plasma Glucose, Glycated Hemoglobin A1c, and 2-Hour Plasma Glucose During 75-g Oral Glucose Tolerance Test According to Sex, Age, and Body Mass Index

	FPG: 5.6–6.9 mmol/L		HbA1c: 5.7–6.4%		2-h PG OGTT: 7.8–11.0 mmol/L	
	Prediabetes, % (n/Total)	aPR* (95% CI)	Prediabetes, % (n/Total)	aPR* (95% CI)	Prediabetes, % (n/Total)	aPR* (95% CI)
**Total population**	47.9 (588/1228)	–	31.0 (355/1144)	–	36.8 (57/155)	–
**Sex**						
Male	44.1 (271/614)	1.00 (Reference)	33.4 (187/560)	1.00 (Reference)	39.3 (35/89)	1.00 (Reference)
Female	51.6 (317/614)	1.13 (1.01–1.26)	28.8 (168/584)	0.78 (0.67–0.91)	33.3 (22/66)	0.89 (0.62–1.29)
*P*-value^†^	0.009	–	0.042	–	0.332	–
**Age (years)**						
≤34	41.2 (126/306)	1.00 (Reference)	15.1 (42/279)	1.00 (Reference)	32.5 (13/40)	1.00 (Reference)
35–44	45.4 (153/337)	1.06 (0.88–1.26)	23.9 (76/318)	1.56 (1.11–2.19)	25.0 (13/52)	0.82 (0.44–1.53)
45–54	49.3 (188/381)	1.23 (1.04–1.45)	40.2 (140/348)	2.74 (2.02–3.73)	46.8 (22/47)	1.65 (1.01–2.70)
≥55	59.3 (121/204)	1.50 (1.26–1.78)	48.7 (97/199)	3.35 (2.46–4.58)	56.3 (9/16)	1.69 (0.99–2.90)
*P*-value^†^	< 0.001	–	< 0.001	–	0.008	–
**BMI**						
Under/normal weight ^‡^	37.6 (88/234)	1.00 (Reference)	19.3 (42/218)	1.00 (Reference)	25.0 (7/28)	1.00 (Reference)
Overweight	48.1 (259/538)	1.27 (1.05–1.52)	31.1 (158/508)	1.34 (1.00–1.79)	28.8 (19/66)	1.10 (0.53–2.28)
Obese	52.9 (241/456)	1.40 (1.17–1.69)	37.1 (155/418)	1.62 (1.21–2.16)	50.8 (31/61)	1.84 (0.93–3.63)
*P*-value^†^	< 0.001	–	< 0.001	–	0.007	–

**Notes:** *Adjusted for all variables shown in Table; ^†^calculated using chi-squared tests. Fisher’s exact test was used to calculate p-values when the cell counts were <5; ^‡^the underweight group (body mass index <18.5) was combined with the normal weight group (body mass index between 18.5% and 24.9%), because only 18 (1.5%) study participants were classified as underweight.

**Abbreviations:** BMI, body mass index; FPG, fasting plasma glucose; HbA1c, glycated hemoglobin A1; PG, plasma glucose; OGTT, oral glucose tolerance test; aPR, adjusted prevalence ratio; CI, confidence interval.

The proportion of undiagnosed T2DM defined according to FPG was similar between males and females (7.2% vs 6.7%, *P* = 0.717; Table 3). In contrast, more males were identified as having undiagnosed T2DM than females according to HbA1c criteria (6.3% vs 3.6%, *P* = 0.018). Similarly, the 2-h PG OGTT suggested that more males had undiagnosed T2DM than females (12.4% vs 9.1%, *P* = 0.432), although this difference was not significant. Prevalence estimates of undiagnosed T2DM demonstrated increasing trends across the age and BMI groups (Table 3). For example, the prevalence of HbA1c-defined undiagnosed T2DM increased from 0.9% among individuals categorized as under/normal weight to 9.6% among subjects classified as obese according to their BMI (aPR = 8.09, 95% CI: 2.00–32.73). Moreover, the prevalence of HbA1c-defined prediabetes was highest among participants aged ≥55 years compared to those aged ≤34 years (aPR = 19.67, 95% CI: 4.75–81.58; Table 3).

**Table 3:** Prevalence Estimates of Undiagnosed Type-2 Diabetes Mellitus Measured by Fasting Plasma Glucose, Glycated Hemoglobin A1c, and 2-Hour Plasma Glucose During 75-g Oral Glucose Tolerance Test According to Sex, Age, and Body Mass Index

	FPG: ≥ 7.0 mmol/L		HbA1c: ≥ 6.5%		2-h PG OGTT: ≥ 11.1 mmol/L	
	Undiagnosed T2DM, % (n/Total)	aPR* (95% CI)	Undiagnosed T2DM, % (n/Total)	aPR* (95% CI)	Undiagnosed T2DM, % (n/Total)	aPR* (95% CI)
**Total population**	6.9 (85/1228)	–	4.9 (56/1144)	–	11.0 (17/155)	–
**Sex**						
Male	7.2 (44/614)	1.00 (Reference)	6.3 (35/560)	1.00 (Reference)	12.4 (11/89)	1.00 (Reference)
Female	6.7 (41/614)	0.88 (0.61–1.26)	3.6 (21/584)	0.43 (0.27–0.69)	9.1 (6/66)	0.68 (0.27–1.71)
*P*-value^†^	0.717	–	0.018	–	0.432	–
**Age (years)**						
≤34	0.7 (2/306)	1.00 (Reference)	0.7 (2/279)	1.00 (Reference)	7.5 (3/40)	1.00 (Reference)
35–44	3.0 (10/337)	4.35 (0.96–19.73)	2.2 (7/318)	3.24 (0.70–15.07)	7.7 (4/52)	0.91 (0.23–3.57)
45–54	11.8 (45/381)	17.61 (4.30–72.04)	7.5 (26/348)	13.47 (3.28–55.35)	14.9 (7/47)	2.51 (0.78–8.10)
≥55	13.7 (28/204)	24.11 (5.82–99.91)	10.6 (21/199)	19.67 (4.75–81.58)	18.8 (3/16)	3.04 (0.84–11.00)
*P*-value^†^	< 0.001	–	< 0.001	–	0.065	–
**BMI**						
Under/normal weight^‡^	2.1 (5/234)	1.00 (Reference)	0.9 (2/218)	1.00 (Reference)	3.6 (1/28)	1.00 (Reference)
Overweight	6.3 (34/538)	1.97 (0.79–4.88)	2.8 (14/508)	2.38 (0.56–10.06)	13.6 (9/66)	2.91 (0.41–20.53)
Obese	10.1 (46/456)	3.28 (1.33–8.08)	9.6 (40/418)	8.09 (2.00–32.73)	11.5 (7/61)	3.76 (0.45–29.23)
*P*-value^†^	< 0.001	–	< 0.001	–	0.207	–

**Notes:** ^†^Calculated using chi-squared tests; Fisher’s exact test was used to calculate p-values when the cell counts were <5; ^‡^the underweight group (body mass index <18.5) was combined with the normal weight group (body mass index between 18.5% and 24.9%), because only 18 (1.5%) study participants were classified as underweight; *Adjusted for all variables shown in Table.

**Abbreviations:** T2DM, type 2 diabetes mellitus; BMI, body mass index; FPG, fasting plasma glucose; HbA1c, glycated hemoglobin A1; PG, plasma glucose; OGTT, oral glucose tolerance test; aPR, adjusted prevalence ratio; CI, confidence interval.

## Discussion

This cross-sectional study estimated the prevalence of prediabetes and undiagnosed T2DM according to FPG, HbA1c, and 2-h PG OGTT among a sample of Kuwaiti adults. The prevalence of prediabetes and undiagnosed T2DM varied according to the test type, with prediabetes prevalence as high as 47.9% according to FPG criteria, and undiagnosed T2DM was as high as 11.0% according to 2-h PG OGTT. Moreover, the prevalence of FPG-defined prediabetes was more common among females than among males, whereas prediabetes identified by HbA1c and 2-h PG during OGTT was more common among males than females. Sex variations in the prevalence of undiagnosed T2DM were observed, with more males than females being identified by HbA1c and 2-h PG during OGTT. In general, prevalence estimates of prediabetes and undiagnosed T2DM showed increasing trends as age and BMI increased.

The burden of prediabetes in Kuwait has rarely been investigated. A prior investigation conducted among adolescents aged 14 to 19 years in Kuwait estimated the prevalence of HbA1c-defined prediabetes to be 33.3% (95% CI: 31.2%–35.4%) according to the ADA criterion (ie, 5.7 ≤ HbA1c% ≤ 6.4).^12^ A study using the World Health Organization’s (WHO) STEPwise survey methodology estimated the prevalence of prediabetes to be 19.4% (95% CI: 17.9–21.0%) in 2014 among a sample of Kuwaiti adults.^14^ The aforementioned study ascertained prediabetes according to either the ADA HbA1c criterion or the WHO FPG range of 6.1–6.9 mmol/L. Our prediabetes estimates, according to FPG (47.9%), HbA1c (31.0%), and 2-h PG OGTT (36.8%), exceeded the reported estimate in the STEPwise survey of 19.4%. One factor that might partly explain this discrepancy is that participants in the STEPwise survey were, on average, younger (mean age: 36.4 years) than subjects in our study sample (mean age: 43.0 years). Among a sample of Qatari adults, prediabetes prevalence was estimated to be 66% according to IGT and/or impaired fasting glucose (IFG), and 45% met the ADA HbA1c criterion for prediabetes.^20^ Similarly, elevated estimates of prediabetes prevalence have been reported among adults in the United States (37.5%, mean age: 47.5 years)^8^ and China (35.7%, mean age: 43.5 years).^9^ Moreover, a study among adults aged 25–64 years in the Czech Republic reported the prevalence of prediabetes (defined according to the ADA HbA1c criterion) to be 27.8%, which is close to our estimate.^21^ The aforementioned findings among adults in Qatar, the US, and China further corroborate the elevated prediabetes prevalence estimates reported in our study.

In the current study, the prevalence of undiagnosed T2DM varied according to the applied test, with estimates being 11.0% by 2-h PG OGTT, 6.9% by FPG, and 4.9% by HbA1c. A previous study conducted among a sample of working adults in Kuwait in 2007 estimated the prevalence of undiagnosed T2DM to be 4.1% (95% CI: 2.7–6.1%) according to FPG.^11^ A recent meta-analysis on the prevalence of undiagnosed T2DM in countries in the Eastern Mediterranean Region (EMRO) reported a pooled-prevalence estimate of 5.45% (95% CI: 4.77–6.13), which was based on data extracted from 50 studies.^22^ At a global scale, the prevalence of undiagnosed T2DM was reported to be 4.6% among adults in the US,^8^ 6.9% among adults in China,^9^ 1.7% among adults participating in the English Longitudinal Study of Ageing,^23^ 5.37% [meta-analysis pooled-prevalence] among adults living in countries in Africa,^24^ 1.7% among adults living in France,^25^ and 3.4% among adults living in a semi-rural setting in Catalonia, Spain.^26^ Hence, such results indicate the presence of variability in the prevalence of undiagnosed T2DM across nations. Moreover, global estimates have shown that around 50.1% of people living with DM are unaware of their condition, with this estimate being as high as 84.3% in low- and middle-income countries.^1^ In Kuwait, a prior study reported that 41.5% of those with DM are unaware of their condition.^14^ A prior study has demonstrated the criticality of undiagnosed T2DM by showing that subjects with undiagnosed T2DM had poorer cardiovascular profiles than those with diagnosed T2DM.^27^ Hence, given the magnitude of undiagnosed T2DM and the related health and economic burden, public health strategies are needed to mitigate this issue.

Prevalence estimates of prediabetes and undiagnosed T2DM showed increasing trends as age and BMI increased in the current study. Such observations are consistent with prior reports that showed elevated prevalence estimates of prediabetes and undiagnosed T2DM among obese and older individuals.^9^,^12^,^14^,^23^,^28–30^ As for sex differences, our study results showed that HbA1c-defined prediabetes and undiagnosed T2DM prevalence estimates were higher among males compared to females. These results are concomitant with results from the National Health and Nutrition Examination Survey (NHANES) 2011–2014 study, which showed that men are more likely than women to have prediabetes and undiagnosed T2DM.^31^ Similarly, our results showed that prevalence estimates of prediabetes and undiagnosed T2DM defined by 2-h PG OGTT were higher among males than females, although these differences were not significant due to the limited sample size. However, the FPG-defined prediabetes prevalence estimate was higher among females (51.6%) than among males (44.1%). Similar to our findings, a higher average of FPG in women compared to men has been shown in a previous study by Veghari et al, where the FPG values correlated with higher waist circumference.^32^ In our study, the average BMI was higher amongst women 29.2 kg/m^2^ than men 28.2 kg/m^2^, which corroborate with the observed higher prevalence of prediabetes. This observation contradicts prior observations, which showed that prediabetes defined by FPG is higher in males than in females.^33^,^34^ Females tend to store fat in subcutaneous adipose tissue compared with men who demonstrate higher visceral adipose tissue levels. However, females present higher levels of lipids in leg skeletal muscles but without deleterious consequences on insulin sensitivity.^35^ As such, may be affecting the FPG levels in our study population, thus, this observation warrants further corroboration.

Prevalence estimates of both prediabetes and undiagnosed T2DM in the current study demonstrated test-type differences. FPG, 2-h PG 75-g OGTT, and HbA1C are equally appropriate for diagnostic screening.^36^ FPG is an indicator of concurrent glucose levels in the blood after a period of fasting (usually at least 8 h of fasting), whereas the OGTT monitors the tolerance and response of the islets to glucose after an induced glucose load. In comparison, the HbA1C test is influenced by the concentration of glucose in the blood. Since the lifespan of erythrocytes is 120 days, HbA1c reflects the average glucose concentration over the preceding 8–12 weeks.^37^ Therefore, the observed variations in the prevalence of prediabetes can be due to the sensitivity and/or specificity of the test, in addition to the individual’s characteristics, where studies have shown that HbA1c levels are influenced by race and ethnicity.^38^ On the other hand, it has been shown that HbA1c is more specific but less sensitive than OGTT in diagnosing T2DM.^39–41^ Nevertheless, there is no evidence that one test should be preferred to diagnose T2DM.^40^ For instance, in the DETECT-2 study (~45,000 participants), neither the OGTT, HbA1c, nor FPG showed an advantage over the others in identifying diabetes-specific retinopathy.^42^ As for undiagnosed T2DM, data from the NHANES study showed that the prevalence of undiagnosed T2DM differed according to the type of the test, with the highest estimate identified by 2-h PG OGGT (3.3%) and the lowest by HbA1C (1.9%), with FPG falling in between (2.1%).^36^ Therefore, the observed test-type differences in prevalence estimates in the current report are in agreement with the existing literature.

The strengths of the current study include the large and representative study sample that allowed us to estimate the prevalence of prediabetes and undiagnosed T2DM among Kuwaiti adults. Moreover, estimating the prevalence according to three tests adds to the strength of our study. Nonetheless, our study has some limitations. Only 12.5% (155/1238) of the total study participants agreed to undergo an OGTT. However, there were no differences between the total study sample and the subsample that participated in the OGTT with regard to age or BMI. Hence, self-selection bias is not a major concern. Prevalence estimates based on 2-h PG OGTT should be interpreted with caution due to the limited sample size. Moreover, given the limited number of participants who underwent OGTT, the results of association analyses in the subsample of participants with OGTT information may be statistically underpowered to detect statistically significant associations. The wide CIs observed for the effect measures relating obesity with undiagnosed T2DM (Table 3) indicate that our study did not have the statistical power to detect such associations with a high degree of precision. Nevertheless, associations between obesity and undiagnosed T2DM, defined according to FPG and HbA1c levels, demonstrated statistical significance. Information bias is inevitable in epidemiologic studies; however, the use of different objective tests to ascertain prediabetes and undiagnosed T2DM in the current report helped to minimize the effect of misclassification and allowed us to compare across test types. Moreover, we have assumed that those fulfilling the undiagnosed DM criteria suffered from T2DM and not T1DM, as the latter is associated with more progressive symptoms, hence leading to early identification, and usually is developed during early stages of life.^43^ The average BMI of participants in our study (males: 28.2 kg/m^2^; women: 29.2 kg/m^2^) is similar to a prior study conducted among Kuwaiti adults (males: 28.4 kg/m^2^; females: 29.1 kg/m^2^; n = 3,589).^44^ This further indicates that our study sample is representative of the Kuwaiti population in terms of BMI, which is a main risk factor for diabetes and prediabetes. Moreover, such an observation indicates that the findings of our study are not biased due to self-selection bias related to the exposure of interest (ie, obesity). In addition, the sex distribution of our study participants closely resembled the population-level distribution.

## Conclusion

The current study showed that a large proportion of Kuwaiti adults are affected by prediabetes, and a considerable proportion of the general population fulfilled the definition of undiagnosed T2DM. Moreover, prevalence estimates of prediabetes and undiagnosed T2DM demonstrated increasing trends as age and BMI increased. Hence, older and obese individuals are more vulnerable to DM-related complications. Given that prediabetes and undiagnosed T2DM are associated with poor microvascular and macrovascular complications, early detection and management of affected individuals through lifestyle modifications and pharmacological treatments may help to reduce the public health burden of these conditions and improve individuals’ health.

## Data Sharing Statement

The data used to support the findings of this study are available from the corresponding author upon request.
